# Looking Beyond the Hip: Multisystem Symptoms and Reversibility Following Revision of Metal-on-Metal Total Hip Arthroplasty

**DOI:** 10.7759/cureus.103461

**Published:** 2026-02-12

**Authors:** Zachary Paragas, Lucas Kasson, Nicholas K Pappa, Douglas Chonko

**Affiliations:** 1 Orthopaedic Surgery, The Ohio State University College of Medicine, Columbus, USA; 2 Orthopaedic Surgery, The Ohio State University Wexner Medical Center, Columbus, USA

**Keywords:** autoimmune, autoinflammatory, metallosis, osteoarthritis, revision total joint arthroplasty, total hip arthroplasty

## Abstract

Metal-on-metal total hip arthroplasty is associated with adverse local tissue reactions and, less commonly, systemic manifestations related to metal debris, though the reversibility of these effects following revision arthroplasty remains incompletely defined. We present the case of a male patient who underwent uncemented metal-on-metal total hip arthroplasty with initially favorable outcomes but developed progressive hip pain and multisystem symptoms more than a decade later, including neuropsychiatric and dermatologic manifestations evaluated across multiple specialties. Imaging demonstrated a large periprosthetic pseudotumor with extensive osteolysis. The patient underwent revision arthroplasty with removal of the metal articulation and extensive debridement, resulting in marked improvement in hip pain and complete resolution of longstanding neuropsychiatric and dermatologic symptoms without changes to psychiatric medications. In contrast, his pre-existing nonischemic cardiomyopathy progressed despite revision, ultimately necessitating cardiac transplantation. This case highlights the diagnostic complexity of metal-on-metal arthroplasty-related complications, the limitations of serum metal-ion testing, and the distinction between reversible systemic manifestations and irreversible end-organ disease, supporting the importance of long-term surveillance and individualized patient counseling.

## Introduction

Metal-on-metal total hip arthroplasty (THA) re-emerged in the late 1990s as a second-generation bearing technology intended to address concerns regarding polyethylene wear and aseptic loosening associated with conventional metal-on-polyethylene constructs, particularly in younger and more active patient populations [1]. These implants were designed to produce lower volumetric wear and permitted the use of larger femoral head diameters, which were believed to confer improved joint stability and increased range of motion [1,2].

The use of metal-on-metal prostheses peaked around 2008, when they accounted for approximately 21% of all hip replacements in the UK and represented over 35% of total hip arthroplasty implants globally [2,3]. Between 2006 and 2012, more than 600,000 metal-on-metal total hip arthroplasty procedures were performed in the United States alone [4]. However, by 2008-2010, registry data began to reveal unacceptably high failure rates compared to other bearing surfaces [1,2,5]. The high failure rates observed with metal-on-metal hip arthroplasty were largely attributed to adverse reactions to metal debris, in which metal particles and ions generated at the bearing surface deposit in surrounding tissues (metallosis) and provoke local inflammatory and immunologic responses. These reactions can lead to progressive osteolysis, soft-tissue necrosis, non-neoplastic inflammatory soft-tissue masses (pseudotumors), and large sterile effusions, ultimately compromising implant stability and surrounding soft tissues [2,6]. This spectrum of local pathology is commonly described as an adverse local tissue reaction (ALTR), an immune-mediated response to metal wear debris.

In addition to local periprosthetic sequelae, exposure to cobalt and chromium ions may produce systemic toxicity. Cobalt, in particular, can interfere with mitochondrial oxidative phosphorylation, promote oxidative stress, and disrupt cellular enzymatic function, leading to multi-organ effects. Systemic cobalt toxicity has been associated with cardiomyopathy, peripheral neuropathy, cognitive and neuropsychiatric changes, visual and auditory disturbances, thyroid dysfunction, and dermatologic manifestations [7-9]. While the true prevalence is difficult to determine, systemic symptoms have been reported in a minority of patients with elevated metal ion levels, with estimates in the literature suggesting clinically significant systemic involvement in a small but meaningful subset of affected individuals [7-9]. These presentations often develop insidiously and may prompt evaluation across multiple specialties before an orthopedic etiology is recognized.

Despite growing recognition of adverse local tissue reactions and systemic manifestations associated with metal-on-metal total hip arthroplasty, important gaps remain in the literature regarding the breadth of clinical presentations, diagnostic complexity, and potential reversibility of associated symptoms. Prior studies have predominantly emphasized isolated organ system toxicity, implant-specific failure mechanisms, or correlations between metal ion levels and adverse outcomes [7-10]. In contrast, comparatively few reports have described patients with extensive periprosthetic disease and multisystem symptoms assessed across multiple specialties, or have examined the extent to which these manifestations may improve following revision arthroplasty.

We report the case of a patient with a history of metal-on-metal total hip arthroplasty who developed progressive symptoms prompting multidisciplinary evaluation and eventual revision surgery. This case contributes to the limited literature addressing symptom reversibility after removal of the metal articulation and underscores the challenge of distinguishing potentially reversible systemic effects from irreversible disease processes.

Study objectives

(1) Describe the clinical presentation, diagnostic workup, and multidisciplinary evaluation of a patient with metal-on-metal total hip arthroplasty who developed extensive adverse local tissue reaction and multisystem symptoms. (2) Review the pathophysiologic mechanisms linking metal debris exposure, metallosis, and cobalt toxicity to both local tissue destruction and systemic manifestations. (3) Highlight the potential for symptom improvement following revision arthroplasty and emphasize the diagnostic challenges in distinguishing reversible metal-related toxicity from irreversible disease processes.

## Case presentation

A 42-year-old male initially presented to a private practice joint replacement clinic in 2008 with progressive left hip pain consistent with symptomatic osteoarthritis. Radiographic evaluation demonstrated degenerative changes of the left hip, and the patient subsequently underwent an uncemented Biomet metal-on-metal left total hip arthroplasty in 2009 for degenerative joint disease.

Following the primary total hip arthroplasty, the patient experienced excellent early outcomes with uncomplicated orthopedic follow-up through 2017.

The patient's complex cardiac history predated the primary arthroplasty and included nonischemic cardiomyopathy with a left ventricular ejection fraction of 30-35%, initially well managed with guideline-directed medical therapy. Over subsequent years, his cardiac function fluctuated, with serial imaging demonstrating ejection fractions ranging from <15% to 35%. Despite optimal medical management, progressive systolic dysfunction necessitated implantation of a biventricular implantable cardioverter-defibrillator in 2017, which subsequently identified episodes of paroxysmal atrial fibrillation requiring long-term anticoagulation.

Beginning in 2021, both the patient and his spouse noted the gradual onset of worsening fatigue, cognitive “cloudiness,” anxiety, and depressive symptoms. These symptoms were managed longitudinally by psychiatry with behavioral and pharmacologic interventions. Over time, the patient’s neuropsychiatric symptoms waxed and waned and frequently demonstrated limited or transient response to multiple well-supported pharmacologic therapies, including bupropion, aripiprazole, buspirone, divalproex, and lorazepam. He underwent longitudinal evaluation by psychiatry and primary care, with medication adjustments and behavioral therapy, without a clear alternative medical or neurologic etiology identified. During this period, the patient also developed dermatologic issues, including persistent inflammatory facial and scalp acne characterized by erythematous papules and pustules, which were initially attributed to divalproex therapy but failed to resolve following medication discontinuation. Dermatologic symptoms persisted despite medication cessation and routine outpatient management, without identification of another clear precipitating cause.

In April 2023, at age 56, the patient presented for orthopedic re-evaluation of worsening left hip pain approximately 14 years after the index total hip arthroplasty. He reported an insidious onset of left groin and lateral hip pain that progressively worsened and ultimately interfered with activities of daily living and recreational travel.

**Figure 1:**
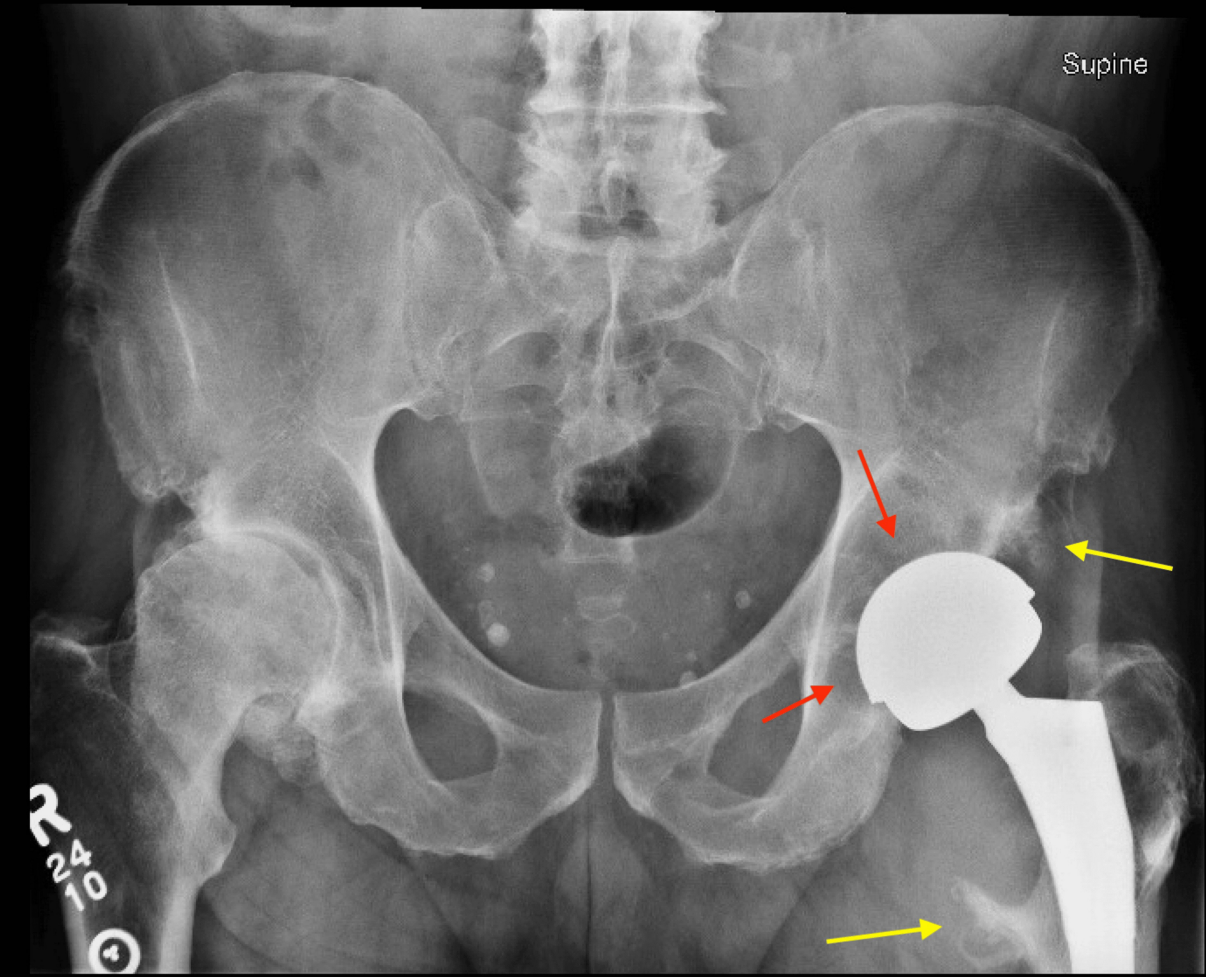
Anterior-posterior radiograph of the left hip demonstrating postsurgical changes consistent with prior metal-on-metal total hip arthroplasty. Red arrows: Patchy periprosthetic lucencies surrounding the acetabular component; Yellow arrows: Enthesopathic changes at the lesser trochanter and along the anterior inferior iliac spine.

Radiographs demonstrated a metal-on-metal left total hip arthroplasty with progressive periprosthetic lucency and osteolysis involving the acetabulum and proximal femur, including bone loss near the lesser trochanter (Figure 1). Laboratory evaluation revealed mildly elevated inflammatory markers and insignificant metal ion testing (Table 1).

**Table 1:** Laboratory findings at orthopedic hip re-evaluation; collected in April 2023.

Parameter (unit)	Value	Normal range
WBC (K/mcL)	9.4	4.6-10.2 K/mcL
C-reactive protein (mg/dL)	2.9	0.0-1.0 mg/dL
Erythrocyte sedimentation rate (mm/hr)	45	0-15 mm/hr
Chromium, serum (mcg/L)	0.4	≤1.4 mcg/L
Cobalt, serum (µg/L)	<1.0	≤1.0 µg/L
Nickel (µg/L)	<2.0	≤10.0 µg/L
Titanium (µg/L)	Undetectable	<5 mcg/L

Hip aspiration demonstrated an elevated synovial white blood cell count of 10,220 cells/µL, with negative aerobic, anaerobic, fungal, and acid-fast cultures.

**Figure 2:**
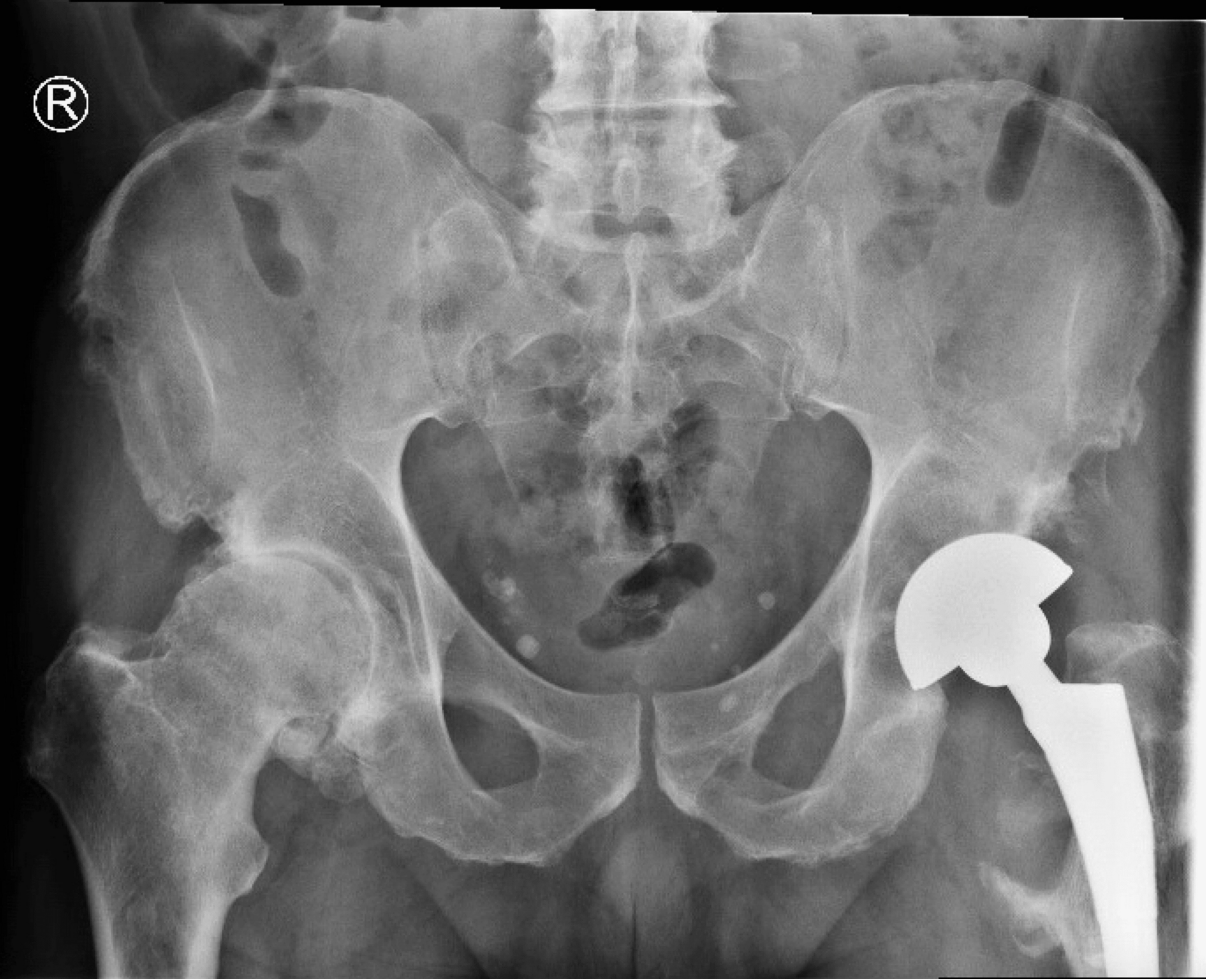
Anterior-posterior pelvis radiograph of revised left total hip arthroplasty with polyethylene liner and ceramic head.

Given concern for severe metallosis and adverse local tissue reactions (ALTRs), the patient underwent revision left total hip arthroplasty in July 2023. Intraoperatively, a large pseudotumor with extensive metallosis was encountered, and approximately one pint of necrotic and inflammatory tissue was excised. Severe osteolysis was present; however, the femoral stem and acetabular shell were well-fixed and retained. The metal-on-metal articulation was revised to a dual-mobility construct with a polyethylene liner and a ceramic head, followed by extensive irrigation and debridement. Post-operative films illustrated a stable left total hip arthroplasty (Figure 2).

Postoperatively, the patient experienced rapid and sustained improvement in hip pain, gait, and function. At six weeks, he reported no pain and improved strength and balance. 

At psychiatric follow-up approximately two months postoperatively, both the patient and his spouse reported a marked and sustained improvement in neuropsychiatric symptoms that had previously been present for several years. The patient described near-complete resolution of cognitive “cloudiness,” anxiety, and depressive symptoms, with restoration of baseline concentration, affect, and emotional regulation. He reported feeling “like a new man,” citing renewed motivation, improved energy levels, and a substantial improvement in overall quality of life. Notably, these improvements were observed in the absence of changes to his psychiatric medication regimen. Although standardized psychiatric symptom scales were not collected pre- or postoperatively, the improvement was consistently documented across follow-up visits and corroborated by both the patient and his spouse.

In addition to the improvement in mood and cognition, the patient and his spouse reported spontaneous resolution of chronic facial and scalp acne that had persisted for years despite prior medication adjustments and dermatologic management. No new medications, topical treatments, or changes in hygiene practices were introduced during this period. The temporal association between revision surgery and resolution of both neuropsychiatric and dermatologic symptoms was noted on serial clinical follow-up, though objective dermatologic scoring measures were not obtained.

Approximately eight months following the revision THA, the patient developed progressive cardiac decompensation related to recurrent atrial arrhythmias, culminating in cardiogenic shock. He ultimately underwent orthotopic heart transplantation in June 2024. His postoperative course was complicated by renal failure requiring hemodialysis, heparin-induced thrombocytopenia, and intracranial hemorrhage, and he passed away following transition to comfort-focused care.

## Discussion

This case illustrates the complex and often delayed clinical course of adverse reactions associated with metal-on-metal total hip arthroplasty, highlighting challenges in diagnosis, the breadth of potential systemic manifestations, and the nuanced distinction between reversible and irreversible disease processes. Although ALTR and implant failure mechanisms related to metal debris are now well recognized, the systemic consequences of metal exposure and the extent to which these manifestations may improve following revision arthroplasty remain incompletely characterized in the literature [7,8].

In this case, the patient developed extensive periprosthetic disease more than a decade after implantation, including a massive pseudotumor with severe osteolysis of both the acetabulum and proximal femur. Such findings are consistent with advanced adverse reactions to metal debris, which have been shown to progress insidiously and may remain clinically occult until substantial soft-tissue destruction has occurred [11,12]. Prior studies have emphasized that the severity of ALTR does not reliably correlate with symptom duration or early clinical findings, contributing to delayed diagnosis and presentation in some patients [13]. Furthermore, pseudotumors have been identified in both symptomatic and asymptomatic individuals and may be present even in well-functioning metal-on-metal hips, reinforcing the need for continued vigilance regardless of early clinical status [13]. The degree of periprosthetic pathology observed in this patient, despite relatively late orthopedic re-evaluation, underscores the importance of long-term surveillance in patients with metal-on-metal implants.

Beyond the local periprosthetic pathology, this case is notable for prominent neuropsychiatric and dermatologic symptoms that evolved over several years and were evaluated and treated by multiple specialties. Systemic manifestations of metal debris exposure, including cognitive impairment, mood disturbance, anxiety, dermatologic changes, endocrine dysfunction, and cardiomyopathy, have been described in prior reports, though much of the literature has focused on isolated organ system involvement or extreme cases of cobalt toxicity [7,8]. As a result, the broader clinical phenotype of multisystem involvement occurring concurrently with extensive periprosthetic disease has been less frequently characterized.

Importantly, these findings must be interpreted within an appropriate clinical differential. Alternative etiologies were considered throughout the patient’s course. Dermatologic symptoms were initially suspected to be medication-related, particularly in the setting of divalproex therapy, but persisted despite discontinuation and routine outpatient management. Neuropsychiatric symptoms were evaluated longitudinally by psychiatry and considered in the context of primary mood and anxiety disorders, while cardiac decline was attributable, at least in part, to progression of known nonischemic cardiomyopathy and recurrent arrhythmia-related decompensation. Despite ongoing multidisciplinary evaluation, no single alternative diagnosis fully accounted for the constellation, timing, and progression of dermatologic, neuropsychiatric, and systemic findings. Acknowledging this broader differential is essential to avoid attributing the presentation solely to metal-related toxicity.

Within this context, a particularly compelling aspect of the case was the marked improvement in neuropsychiatric and dermatologic symptoms following revision arthroplasty. Within two months of removal of the metal articulation and extensive debridement, the patient and his spouse reported near-complete resolution of cognitive cloudiness, anxiety, depressive symptoms, and long-standing acne, despite no changes in medications. While several studies have demonstrated improvement following revision of metal-on-metal total hip arthroplasty, the existing literature has largely emphasized pain relief, functional outcomes, and reductions in metal ion levels, with comparatively limited attention to the reversibility of psychiatric, neurologic, dermatologic, or endocrine manifestations [14,15].

While causality cannot be definitively established in individual cases, the close temporal relationship between revision arthroplasty and symptom improvement observed in this patient suggests that at least a subset of systemic manifestations associated with metal-on-metal arthroplasty may be partially or fully reversible following removal of the inciting source. However, the literature addressing systemic symptom reversibility remains limited and is largely composed of case reports and small series, underscoring the need for further investigation in larger, well-characterized cohorts to better define the spectrum, mechanisms, and durability of post-revision recovery.

At the same time, this case highlights the inherent limitations of symptom reversibility in the presence of long-standing comorbid disease. The patient's nonischemic cardiomyopathy clearly predated his arthroplasty and progressed over time to end-stage heart failure despite guideline-directed medical therapy and eventual cardiac transplantation. Although cobalt-associated cardiomyopathy has been described in the context of metal-on-metal arthroplasty, distinguishing metal-related contributions from irreversible myocardial pathology in patients with complex cardiac histories remains challenging [16,17]. In this context, it is difficult to determine whether metal exposure influenced the trajectory of cardiac decline or whether disease progression reflected the natural history of cardiomyopathy. This distinction is clinically important when counseling patients, as metallosis may exacerbate pre-existing conditions and removal of metal debris may alleviate certain systemic manifestations without reversing established end-organ dysfunction.

Finally, this case reinforces the importance of a comprehensive diagnostic evaluation in patients with metal-on-metal hip arthroplasty. Although serum metal ion levels are frequently incorporated into surveillance strategies, prior studies have demonstrated poor correlation between circulating cobalt and chromium concentrations and the severity of adverse local tissue reactions or pseudotumor burden, limiting their utility as standalone screening tools (Table 1) [10]. Accordingly, multiple authorities caution that metal ion testing should be interpreted as an adjunct rather than a definitive measure, as reliance on serum markers alone may delay recognition of clinically significant periprosthetic disease and definitive management [18]. Cross-sectional imaging, therefore, plays a central role in characterizing periprosthetic pathology. In particular, metal artifact reduction sequence magnetic resonance imaging (MARS MRI) is widely used to identify pseudotumors, osteolysis, abductor pathology, and neurovascular involvement, and has been incorporated into major regulatory follow-up recommendations [19,20]. Advanced imaging should thus be strongly considered when patients with metal-on-metal implants present with unexplained hip pain or systemic symptoms, even when laboratory values are inconclusive [20].

## Conclusions

Metal-on-metal total hip arthroplasty may be associated with extensive local tissue destruction and multisystem symptoms that develop insidiously over time. This case suggests that revision arthroplasty with removal of the metal articulation may be followed by meaningful improvement in select systemic manifestations, while established end-organ disease may persist; however, these findings should be interpreted as associative and hypothesis-generating rather than causal. Recognition of these variable outcomes highlights the importance of long-term surveillance, comprehensive diagnostic evaluation, and individualized counseling for patients with metal-on-metal hip implants. Prospective studies incorporating standardized symptom assessment and longitudinal follow-up are needed to better define the systemic effects of metal-on-metal exposure and to clarify the extent to which symptoms may improve after revision.
